# Logistic organ dysfunction system as an early risk stratification tool after aneurysmal subarachnoid hemorrhage

**DOI:** 10.1038/s41598-024-78937-8

**Published:** 2024-11-12

**Authors:** Sheri Tuzi, Beate Kranawetter, Onnen Moerer, Veit Rohde, Dorothee Mielke, Vesna Malinova

**Affiliations:** 1 Department of Neurosurgery, University Medical Center Göttingen, Göttingen, Germany; 2 Department of Anesthesiology, University Medical Center Göttingen, Göttingen, Germany; 3 Department of Neurosurgery, Georg-August-University, Robert-Koch-Straße 40, 37075 Göttingen, Germany; 4 Department of Neurosurgery, University Hospital Augsburg, Augsburg, Germany

**Keywords:** Subarachnoid hemorrhage, Early brain injury, Prognostic tools, Neuroscience, Diseases of the nervous system, Stroke

## Abstract

Aneurysmal subarachnoid hemorrhage (aSAH) not only causes neurological deficits but also influences extracerebral organ functions. The Logistic Organ Dysfunction System (LODS) reliably captures organ dysfunctions and predicts mortality of critically ill patients. This study investigated LODS in the setting of aSAH as a surrogate marker for early brain injury (EBI). Patients with aSAH treated between 2012 and 2020 were retrospectively analyzed. LODS was calculated within 24 h upon admission applying functional parameters for each organ system. The EBI was evaluated based on 1-persistent loss of consciousness, 2-global cerebral edema, and 3-intracranial blood burden. The outcome was assessed with the modified Rankin scale (mRS) at 3-months after ictus (mRS > 2 = unfavorable outcome). A total of 324 patients with a mean age of 55.9 years were included. Severe EBI (EBI grade ≥ 3) was found in 38% (124/324) of patients. Higher LODS score correlated with severe EBI (*p* < 0.0001) and poor outcome (*p* < 0.0001). LODS with a cutoff of 7 allowed a reliable discrimination (AUC 78%, *p* < 0.0001) of patients with severe from those with mild EBI. The LODS-calculation as an early risk stratification and prognostic tool reliably reflected the severity of EBI after aSAH and correlated with outcome.

## Introduction

Aneurysmal subarachnoid hemorrhage (aSAH) is a severe cerebrovascular condition with a high risk for permanent disability^1^. The rupture of an intracranial aneurysm triggers a cascade of pathophysiological processes taking place within the first 72 h following the bleeding event, referred to as early brain injury (EBI)^2^. These processes include cerebral ischemia, inflammation and oxidative stress, disruption of the blood-brain barrier, and electrolyte imbalances^3–6^. Aside from the severity of the initial bleeding, the occurrence of secondary complications during the acute phase mainly contribute to the high morbidity and mortality associated with aSAH^1^. Aneurysmal SAH leads not only to neurological deficits but also has systemic effects, that contribute to the morbidity of aSAH^7^. Clinical grading systems for aSAH are based on the neurological condition of affected patients, while the assessment of extracerebral organ functions remains underrepresented. Several scoring systems are available for the systematic assessment of organ function which enable reliable prediction of outcomes in critically ill patients. The Logistic Organ Dysfunction System (LODS) was developed to assess the severity of organ dysfunction across six organ systems (respiratory, cardiovascular, central nervous, hepatic, renal, and hematological) and to predict the outcomes of ICU patients^8^. The value of LODS as an early risk stratification tool in aSAH patients has not been determined yet, which was the rationale for conducting the study. This study aimed to explore the correlation of LODS with the severity of EBI and with functional outcome after aSAH. The hypothesis was that LODS can reflect the severity of EBI and thus serve as a surrogate marker for EBI.

## Methods

**Fig. 1:**
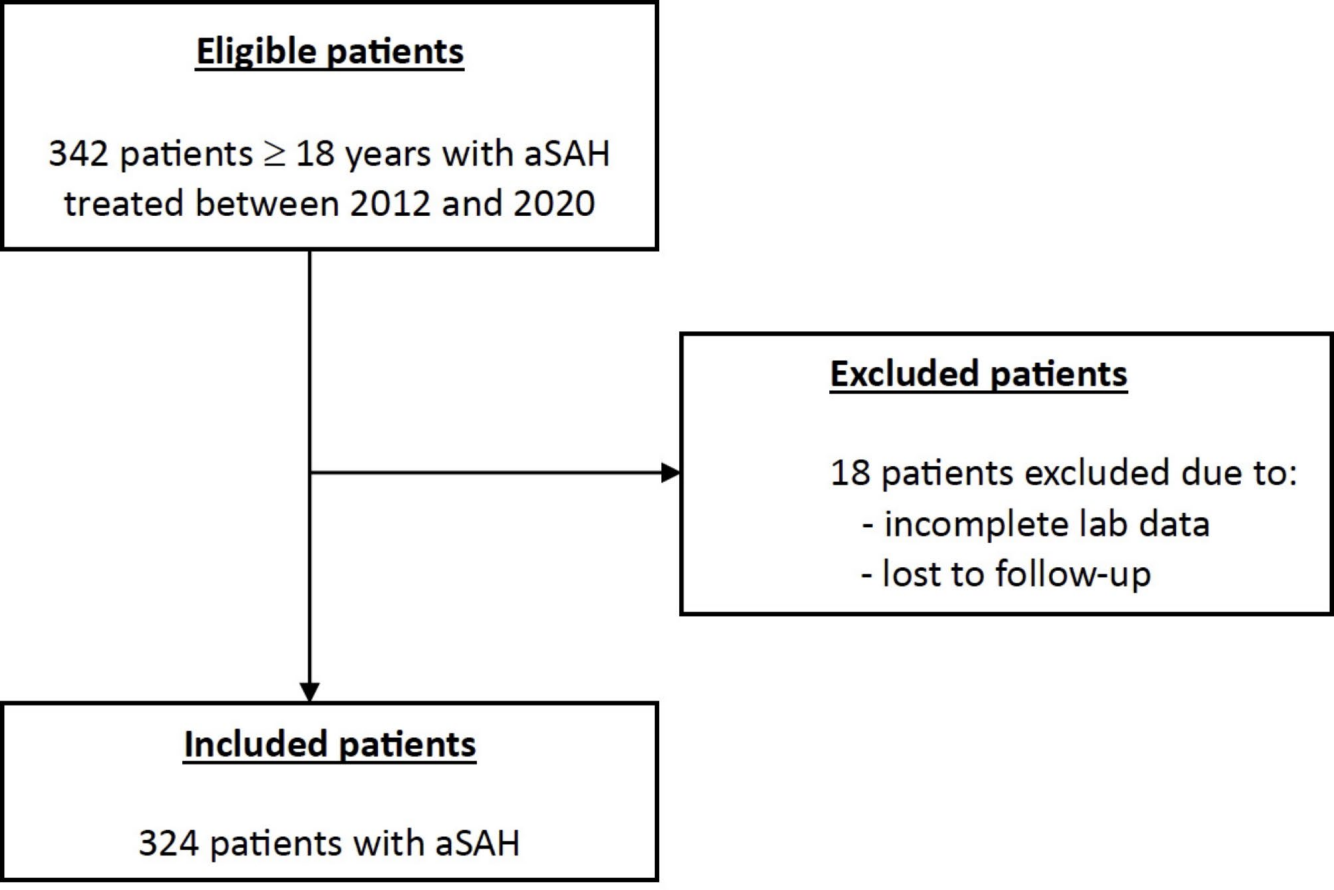
Flow diagram of the study population selection.

In this retrospective observational study, a consecutive patient cohort diagnosed with aSAH and treated at our center from January 2012 to December 2020 was included. The inclusion criteria were adult patients with confirmed aSAH and a complete data documentation concerning the parameters required for the calculation of LODS. The data were complete in the entire patient cohort because all included parameters are evaluated on a regular basis in our clinic. The diagnosis of aSAH was confirmed by cranial computed tomography (CT), computed tomography angiography (CTA), and/or digital subtraction angiography (DSA). Patients with non-aneurysmal SAH were excluded from the study. Only adult patients ≥ 18 years with complete data were enrolled in the study. A flow diagram depicting the study population selection is shown in Fig. 1.

### Calculation of LODS

The LODS was calculated as previously described by Le Gall et al.^8^. A higher LODS score indicates a higher degree of organ dysfunction. For the calculation of LODS, specific physiological and laboratory variables reflecting the function of six major organ systems were extracted from the ICU documentation system. For each variable, points (ranging from 0 to 5, with 0 indicating a normal physiological state and 5 indicating the most severe state) were assigned according to the severity of dysfunction. A summary of considered parameters with the assigned points is given in Table 1. The LODS was calculated including and excluding the Glasgow Coma Scale (GCS) score to separately assess the impact on the extracerebral organ functions.

**Table 1:** Calculation of the logistic organ dysfunction score as described by Le Gall et al.^8^.

Systems	Parameters	5	3	1	0	1	3	5
Neurologic	GCS	3–5	6–8	9–13	14–15	–	–	–
Cardiologic	HR (beats/min)	< 30	–	–	30–139	140	–	–
	SBP (mmHg)	< 40	40–69	70–89	90–239	240–269	≥270	-
Renal	Urea nitrogen (mmol/l)	–	–	–	< 6	6-9.9	10-19.9	> 20
	Creatinine (mg/dl)	–	–	–	< 1.20	1.20–1.59	≥1.60	–
	Urine output (l)	< 0.5	0.5–0.74	–	0.75–9.99	–	≥10	–
Pulmonary	PaO_2_ mmHg/FiO_2_	–	< 150	≥150	No CPAP	–	–	–
	PaO_2_ kPa/FiO_2_	–	< 19.9	≥19.9		–	–	–
Hematologic	Leucocytes (x10^9^/l)	–	< 1.0	1.0-2.4	2.5–49.9	≥50	–	–
	Platelets (10^9^/l)	–	–	–	< 50	≥50	–	–
Hepatic	Bilirubin (mg/dl)	–	–	–	< 2.0	≥2.0	–	–
	PTtime (secs)	–	–	–	≤3	> 3	–	–

The score was calculated by summing the values in the first row of the table.

### Primary and secondary outcome parameters

The severity of EBI was assessed considering the following parameters: 1-persistent loss of consciousness upon admission, 2-generalized cerebral edema, and 3-total intracranial blood burden. To determine the severity, points were assigned as follows: one point for persistent loss of consciousness, one point for global cerebral edema, and additional points based on the assessment of intracranial blood burden using three scoring systems. The Hijdra score^9^ was used to assess subarachnoid blood amount, the Le Roux score^10^ was applied for the assessment of intraventricular blood amount, and the ABC/2 ellipsoid formula^11^ was used for calculation of the intraparenchymal blood volume, with varying points assigned based on the hematoma volume, 1 point for < 10 ml, 2 points for 10–30 ml, and 3 points for > 30 ml. The maximum achievable total intracranial blood burden score is 49, which was further categorized as low (0–19 points), moderate (20–29 points), or high (30–49 points), with corresponding 1, 2, and 3 points assigned. The EBI grade is determined by summing up all the assigned points, resulting in a range of 1 to 5 points. An EBI grade of ≥ 3 was considered as severe EBI. Functional outcome was assessed according to the modified Rankin scale (mRS) at 3 months follow-up. A mRS ≤ 2 was considered as favorable outcome. The Charlson Comorbidity Index (CCI) was calculated as previously described by Charlson et al. to assess comorbidities as possible confounding factors^12^.

### Statistical analysis

The data was analyzed using the GraphPad Prism software (Version 10, GraphPad Software, San Diego, CA, USA) employing both descriptive and inferential statistics. Frequency and percentages were used to represent discrete, ordinal, and binary variables, while continuous variables were expressed as mean ± standard deviation. Correlation and simple logistic regression tests were run for each endpoint with dichotomous variables. For continuous variables, correlation and simple linear regression tests were applied. To display the relationship between sensitivity and specificity and estimate the tests’ overall diagnostic accuracy, and receiver operating characteristic curve (ROC curve) analyses were conducted. A multivariate regression analysis was performed to identify possible confounding factors.

## Results

### Study population

A consecutive patient cohort including 324 patients with aSAH was analyzed. The mean age in the study population was 55.9 ± 13.6 years (range 23–90 years), 64% (207/324) of whom were female, and 36% (117/324) were male. Good clinical grade, WFNS (World Federation of Neurosurgical Societies) I–III was found in 56% (183/324) of the patients. The ruptured aneurysm was treated with microsurgical clipping in 53% (173/324) of cases, and with endovascular coiling in 47% (151/324) of cases. The baseline characteristics of the study cohort are summarized in Table 2.

**Table 2:** Baseline characteristics of the study population.

Variables	Values
Number of patients	324
Mean age (in years)	55.9 ± 13.6 years
Sex	
Male	
Female	64% (207/324)
WFNS grading	
Grade I–III	56% (183/324)
Grade IV–V	44% (141/324)
Fisher grading	
Grade 1–2	9% (30/324)
Grade 3–4	(91%, 294/324)
Aneurysm location	
Anterior circulation	86% (278/324)
Posterior circulation	14% (46/324)
Aneurysm treatment	
Clipping	53% (173/324)
Coiling	47% (151/324)

### LODS vs. EBI severity

**Fig. 2:**
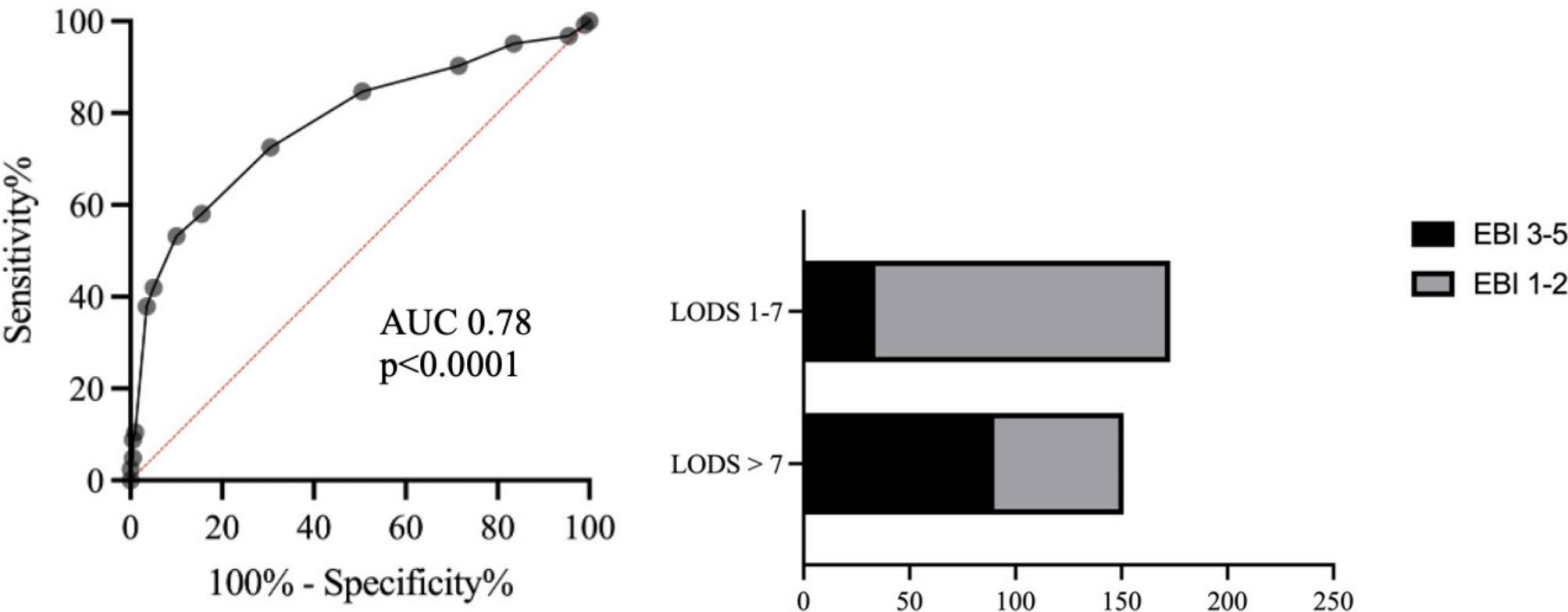
Discrimination power of LODS for differentiating between patients with severe early brain injury (EBI) and those with mild EBI.

**Fig. 3:**
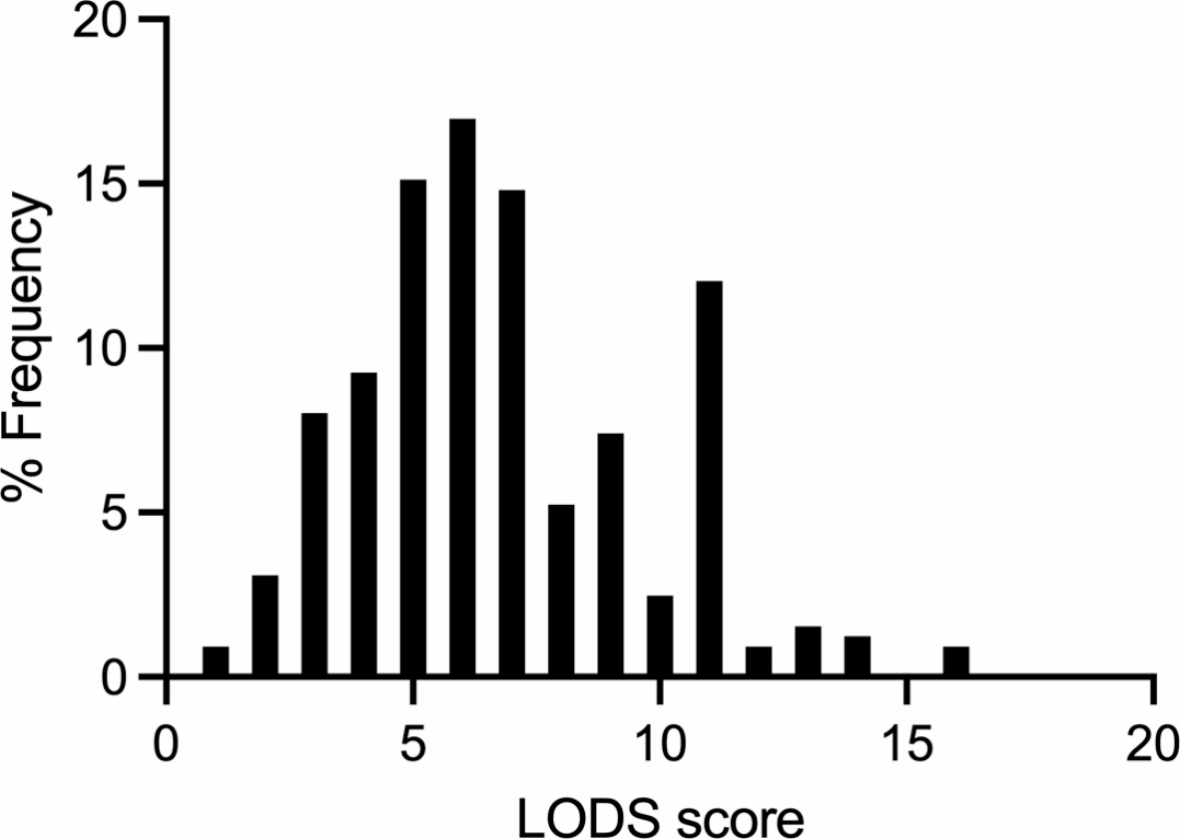
Distribution of the LODS score in the study population with the percentage frequency of every score depicted on the y-axis.

The median EBI grade in the study cohort was 2 (95%CI 1.89–2.11). An EBI grade ≥ 3 was found in 38% (124/324) of patients. The median LODS score (range 0–11) in the study cohort was 4 (95%CI 3.83–4.17). Higher LODS score was significantly associated with severe EBI (*p* < 0.0001, Table 3). LODS with a cutoff value of 7 allowed a reliable discrimination between patients with severe EBI (EBI grade ≥ 3) from those with mild EBI (EBI grade 1–2) with an AUC 78% (95%CI 73–83%), *p* < 0.0001 (Fig. 2). The distribution of the LODS score is depicted in Fig. 3. The probability of severe EBI (EBI grade ≥ 3) was sixfold higher in patients with LODS > 7 compared to those with LODS 1–7 (OR 6.03, 95%CI 3.7–10.3, *p* < 0.0001). A LODS > 7 predicted severe EBI with a sensitivity of 73%, a specificity of 70%, a positive predictive value (PPV) of 60%, and a negative predictive value (NPV) of 80%. The LODS calculated without considering the GCS score was also significantly associated with severe EBI (*p* = 0.0003, Table 3).

**Table 3:** LODS as predictor of EBI severity, functional outcome, and mortality.

Variables	Estimate	Standard error	95% CI	*P* value
LODS as a predictor of EBI severity				
LODS with GCS	3.654	0.292	3.079 to 4.229	< 0.0001*
LODS without GCS	4.522	0.200	4.127 to 4.917	0.0003*
LODS as a predictor of unfavorable outcome (including mortality)				
LODS with GCS	5.768	0.203	5.367 to 6.169	< 0.0001*
LODS without GCS	4.932	0.126	4.683 to 5.181	0.002*
LODS as a predictor of unfavorable outcome (including mortality)				
LODS with GCS	6.691	0.167	6.362 to 7.020	0.01*
LODS without GCS	5.159	0.098	4.966 to 5.352	0.251

### LODS vs. functional outcome and in-hospital mortality

**Fig. 4:**
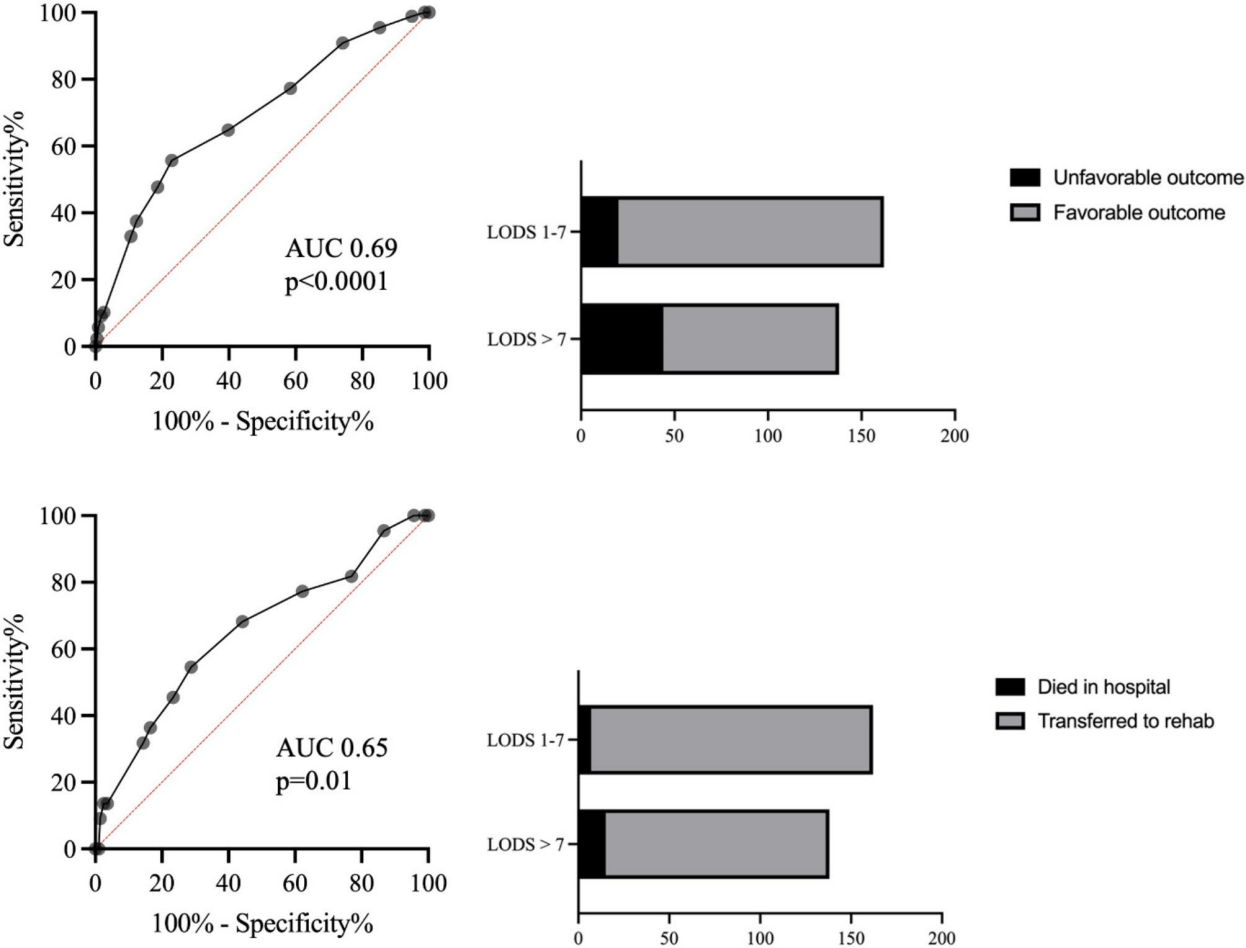
Discrimination power of LODS for differentiating between patients with favorable outcome (mRS ≤ 2) and those unfavorable outcomes including in-hospital mortality (mRS 3–6).

There were 73% (236/324) of patients with favorable outcome. The in-hospital mortality rate was 6.8% (22/324). Higher LODS score significantly correlated with unfavorable outcome according to mRS > 2 (*p* < 0.0001, Table 3) and with in-hospital mortality (*p* = 0.002, Table 3). LODS with a cutoff value of 7 allowed a reliable discrimination between patients with favorable outcome from those with unfavorable outcome with an area under the curve (AUC) 0.69, 95%CI 63–75%, *p* < 0.0001 (Fig. 4). The probability of unfavorable outcome in patients with LODS > 7 was threefold higher compared to those with LODS 1–7 (OR 3.3, 95%CI 1.8-6.0, *p* < 0.0001). A LODS > 7 predicted unfavorable outcomes with a sensitivity of 69%, a specificity of 60%, a PPV of 32%, and a NPV of 88%. LODS with a cutoff value of 7 allowed a reliable discrimination between patients with favorable outcome from those with unfavorable outcome with an AUC 0.65, 95%CI 52–78%, *p* = 0.01 (Fig. 4). The probability of dying in the hospital was twofold higher in patients with LODS > 7 compared to those with LODS 1–7 (OR 2.7, 95%CI 1.1–6.9, *p* = 0.04). A LODS > 7 predicted in-hospital mortality with a sensitivity of 68%, a specificity of 56%, a PPV of 11%, and a NPV of 96%. On the contrary, the LODS without GCS was not significantly associated with the in-hospital mortality (Table 3). In the multivariate regression analysis including possible confounding factors like age, aneurysm treatment modality, and comorbidities (i.e., Charlson Comorbidity Index = CCI) only LODS (*p* = 0.001) and the EBI score (*p* < 0.0001) remained significant predictors of outcome at 3 months follow-up (Table 4). In Table 5 the patients categorized according to EBI score, LODS score, and mRS are shown.

**Table 4:** Multiple regression analysis of outcome predictors at 3 months follow-up.

Variables	Estimate	Standard error	95% CI	ItI	*P* value
Age in years	0.0175	0.0113	– 0.0047 to 0.0398	1.551	0.122
Aneurysm treatment	0.0075	0.1952	– 0.3765 to 0.3916	0.038	0.969
LODS score	0.1314	0.0412	0.05029 to 0.2124	3.188	0.001*
EBI score	0.4541	0.0949	0.2674 to 0.6409	4.784	< 0.0001*
CCI	0.0351	0.0952	– 0.1522 to 0.2226	0.369	0.712

LODS = Logistic Organ Dysfunction System, EBI = early brain injury, CCI = Charlson comorbidity index.

**Table 5:** Categorization of included patients according to EBI score, LODS score, and mRS.

Variables	Values
EBI score % (n)	
1	36.4% (118/324)
2	25.3% (82/324)
3	20.4% (66/324)
4	13.9% (45/324)
5	4% (13/324)
LODS score % (n)	
1	0.9% (3/324)
2	3.1% (10/324)
3	8% (26/324)
4	9.3% (30/324)
5	15.1% (49/324)
6	17% (55/324)
7	15% (48/324)
8	5.2% (17/324)
9	7.4% (24/324)
10	2.5% (8/324)
11	12% (39/324)
12	0.9% (3/324)
13	1.5% (5/324)
14	1.2% (4/324)
15	0% (0/324)
16	0.9% (3/324)
mRS % (n)	
0	35% (113/324)
1	27% (87/324)
2	7.7% (25/324)
3	6.8% (22/324)
4	9.9% (32/324)
5	6.8% (22/324)
6	6.8% (22/324)

EBI = early brain injury, LODS = Logistic Organ Dysfunction System, mRS = modified Rankin Scale.

## Discussion

Aside from neurological dysfunction a systemic reaction with dysfunction of multiple extracerebral organs often occurs after aSAH, especially, in patients with a severe bleeding. Although, several scoring systems have been developed over the years for a systematic assessment of multiple organ functions in critically ill patients, their prognostic value in the setting of aSAH has not been determined yet. In this observational study, the LODS was evaluated in a consecutive patient cohort with aSAH, which showed a strong correlation with the severity of EBI as well as with functional outcome at 3 months follow-up. The LODS was calculated based on parameters, that are available within 24 h upon admission, facilitating an early risk stratification. LODS, with its simplicity and efficiency, offers the potential to enhance resource allocation and clinical decision-making, according to the individual risk for complications. However, to improve accuracy in predicting mortality rates for neurological and neurosurgical illnesses, more robust and specific disease severity scoring methods are needed, ensuring better discrimination and calibration^13^. The correlation between LODS and our EBI grading system indicates that they could complement each other in assessing the severity and prognosis of patients in the early stages after aSAH. Interestingly, the LODS without considering the neurological function as represented by the GCS score was also significantly associated with severe EBI and with unfavorable outcome, but not with the in-hospital mortality. These findings emphasize the importance of considering the extracerebral organ functions in aSAH patients in clinical practice. LODS has been previously confirmed as a prognostic tool for ICU patients with sepsis^14,15^, myocardial infarction^16^, and for patients undergoing cardiac surgery^17^. Furthermore, the LODS outperformed the admission-focused Acute Physiology Assessment and Chronic Health Evaluation (APACHE) II and the Simplified Acute Physiology Score (SAPS) II in predicting outcomes, underscoring the importance of daily postoperative monitoring to assess organ dysfunction development^14^. The authors of previous studies proposed the use of organ dysfunction scores to track a patient’s clinical progress, determine the severity of an illness at a given time, and identify areas for quality improvement^18^. The findings of our study demonstrated for the first-time potential benefits for an early risk stratification in aSAH patients admitted to the ICU with a high negative predictive value for severe EBI and functional outcome as well as in-hospital mortality. The establishment of risk adapted management protocols would allow a better allocation of routine imaging associated with radiation exposure in patients with aSAH warranting a timely detection and treatment of secondary complications in high-risk patients, while avoiding unnecessary radiation exposure due to routine diagnostics in low-risk patients. Additionally, such risk adapted treatment protocols would support the decision-making concerning the duration of observation at the ICU and regarding the required monitoring level in aSAH patients. Despite of several previous studies^19–21^ demonstrating an interaction of extracerebral organ dysfunctions and neurological complications like cerebral vasospasm after aSAH, the role of extracerebral organ functions is still not directly considered during the acute management of the patients. On the one hand, aSAH induces a systemic reaction affecting extracerebral organ functions^20^, and extracerebral organ dysfucntions affect the outcome of patients with aSAH, on the other hand^19,21^. The findings of our study are indicating a need for a more holistic assessment and monitoring of patients admitted with aSAH, rather than looking solely on neurological conditions. With the increasing implementation of clinical systems using artificial intelligence techniques, scores like the LODS can be directly calculated and tracked during the acute management of the patients in the future. Although several scoring systems have been evaluated in the past years for the prediction of functional outcome in aSAH patients, none of these scores could be established to be widely used at the ICU^22–28^. The reason for this may be the consideration of different parameters by different scores and the relatively complex calculation of the scores. The analysis of a large consecutive patient population encompassing 1200 patients several cerebral and extracerebral parameters have been identified as significant predictors of in-hospital mortality after aSAH including higher age, initial loss of consciousness, large aneurysm size, GCS score on admission, modified Fisher score, APACHE II, global cerebral edema, rebleeding, hypotension, pulmonary edema, myocardial ischemia and hepatic failure^25^. The reported cause of death was brain death in 42% and cardiac death in 58%, while 86% of patients with cardiac death hat a do-not-resuscitate (DNR) order^25^. These previous findings show that not only neurological but also extracerebral complications are determinators of outcome highlighting the need for considering cerebral as well as extracerebral parameters for risk stratification concerning the morbidity and mortality after aSAH. Since LODS considers all organ systems including the neurological function, it has the potential of becoming a standard score for use at the ICU as an early risk stratification tool. However, prospective validation and adaptation of the set cutoff values may be necessary to increase the discrimination power of LODS for use in an aSAH patient population.

### Limitations

The retrospective nature of this study introduces inherent limitations, as it relies on historical data and may not account for all variables or provide the same level of control as prospective studies. The tool used in this study was not originally designed or tailored for aSAH patients. This may impact its precision and relevance in this specific patient population. While it is a retrospective analysis, the LODS could represent a valuable early risk stratification tool for aSAH patients treated at the ICU. These preliminary results indicate a need for further research to adapt and refine the tool for aSAH patients, potentially enhancing our ability to assess and manage their critical care needs.

## Conclusions

LODS reliably reflected the severity of EBI in the setting of aSAH and correlated with functional outcome. The calculation of LODS upon admission in aSAH patients could be helpful as a valuable and early risk stratification and a prognostic tool.

## Data Availability

All data are already presented in the manuscript.
